# Exploiting single-molecule transcript sequencing for eukaryotic gene prediction

**DOI:** 10.1186/s13059-015-0729-7

**Published:** 2015-09-02

**Authors:** André E. Minoche, Juliane C. Dohm, Jessica Schneider, Daniela Holtgräwe, Prisca Viehöver, Magda Montfort, Thomas Rosleff Sörensen, Bernd Weisshaar, Heinz Himmelbauer

**Affiliations:** Max Planck Institute for Molecular Genetics, Berlin, Germany; Centre for Genomic Regulation (CRG), Barcelona, Spain; Universitat Pompeu Fabra (UPF), Barcelona, Spain; University of Natural Resources and Life Sciences (BOKU), Muthgasse 18, 1190 Vienna, Austria; Department of Biology/Center for Biotechnology, Bielefeld University, 33615 Bielefeld, Germany

**Keywords:** Eukaryotic gene prediction, Single-molecule real-time sequencing, mRNA-seq, Caryophyllales, Sugar beet, Spinach, Non-model species, Genome annotation

## Abstract

We develop a method to predict and validate gene models using PacBio single-molecule, real-time (SMRT) cDNA reads. Ninety-eight percent of full-insert SMRT reads span complete open reading frames. Gene model validation using SMRT reads is developed as automated process. Optimized training and prediction settings and mRNA-seq noise reduction of assisting Illumina reads results in increased gene prediction sensitivity and precision. Additionally, we present an improved gene set for sugar beet (*Beta vulgaris*) and the first genome-wide gene set for spinach (*Spinacia oleracea*). The workflow and guidelines are a valuable resource to obtain comprehensive gene sets for newly sequenced genomes of non-model eukaryotes.

**Electronic supplementary material:**

The online version of this article (doi:10.1186/s13059-015-0729-7) contains supplementary material, which is available to authorized users.

## Background

Genes hardest to predict correctly with current prediction programs show structures with large numbers of exons, very short exons, long introns, weak translation start signals, non-canonical splice sites, or many isoforms [1]. Gene prediction may be improved by adapting *ab initio* prediction parameters to the genome-specific base composition in coding sequence, splice sites, and intergenic regions. Also, extended expression evidence may support more genes of low expression, or genes expressed only under highly specific physiological conditions. In addition, full-length sequencing of transcripts facilitates the resolution of complex gene structures.

Several technologies exist that allow sequencing of the transcriptome. Most of these technologies are not capable of generating reads representing entire transcripts due to the sequencing-read shortness. Single-molecule, real-time (SMRT) sequencing developed by Pacific Biosciences [2] overcomes this limitation [3] by enabling the generation of kilobase-sized sequencing reads. By employing appropriate methods for cDNA preparation, *bona fide* full-length transcript sequences can be generated. Current SMRT sequencing reads exhibit high sequencing error rates, most notably base insertions or deletions. However, due to the random nature of the encountered errors, the construction of highly accurate consensus by re-iterated sequencing of the same fragment is straightforward.

Caryophyllales are a large clade that currently comprises 11,510 species including the crop plants sugar beet, spinach, and quinoa, as well as cacti, ice plants, and carnivorous plants that have adapted to stressful environments [4]. A few previous studies on Caryophyllales presented annotation of sugar beet genes, including a normalized collection of expressed sequence tag (EST) data [5], manually refined gene models within a short genomic region [6], ESTs arranged along a sugar beet physical map [7], characterization of sugar beet genes in the context of positional cloning projects or gene family analysis [8–11], or a transcriptome-wide but solely cDNA-based assembly of sugar beet genes [12]. The most comprehensive set of gene predictions for Caryophyllales until now has been generated in the course of the sugar beet genome project [13]. These gene models were predicted using the AUGUSTUS software [14, 15]; AUGUSTUS belongs to the most accurate tools for eukaryotic protein-coding gene prediction [1, 16] by integrating *ab initio* and evidence-based gene finding approaches. Here, we searched for strategies to improve the overall accuracy of gene prediction in non-model species, as exemplified by sugar beet and its sister taxon spinach in the current work. Our workflows exploit full-insert transcript sequences as a prerequisite for automated, high-precision coding gene annotation that supersedes the need for manual curation of genes discovered within newly assembled genomes.

## Results and discussion

**Fig. 1:**
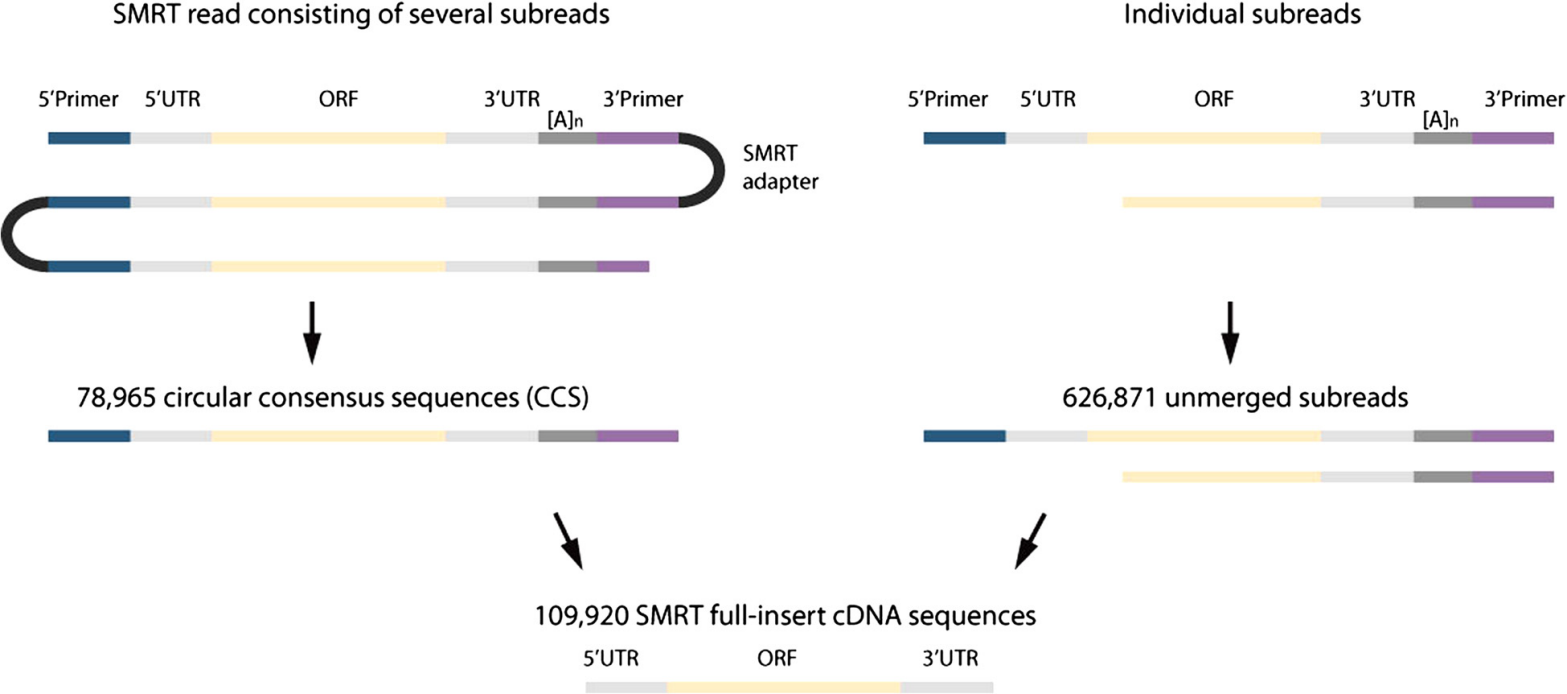
Identification of full-insert cDNA sequences in SMRT sequencing data. Colors refer to the different types of sequences that can be encountered within the read data, that is 5’ and 3’ cDNA synthesis primers, PacBio SMRT library preparation adapter, and cDNA sequences consisting of 5’ UTR, open reading frame (ORF), 3’ UTR, and poly(A) tail. Initially, reads were subclassified into two groups: SMRT reads consisting of several subreads (left) or individual subreads (right). Reads from both groups were error-corrected and used to identify full-length cDNA sequences

**Fig. 2:**
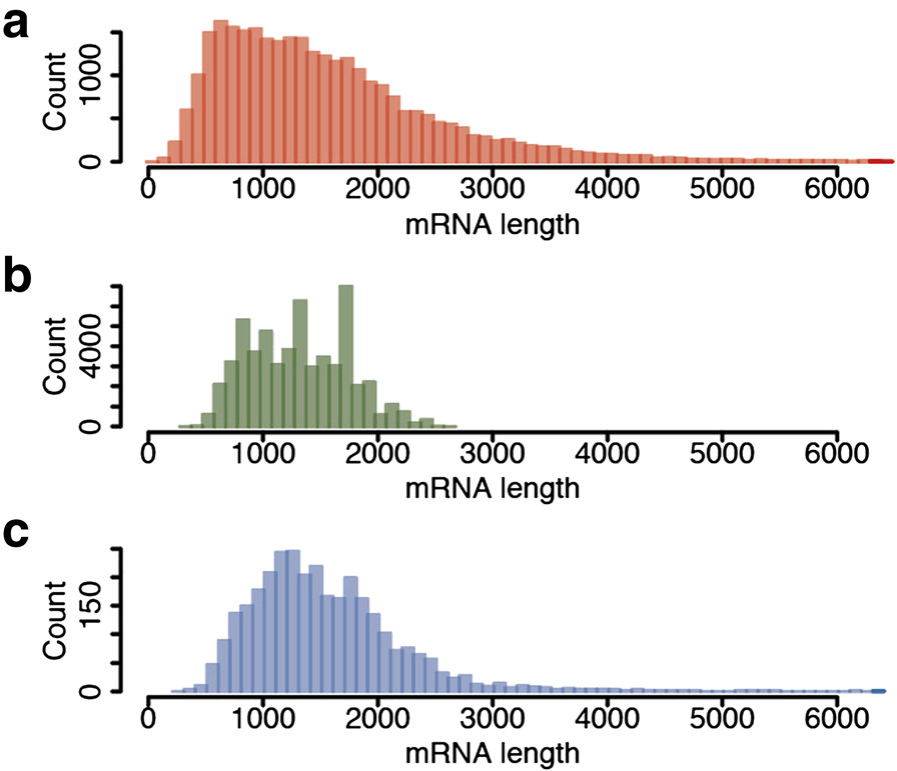
Transcript length distribution. **a** Length distribution of 29,831 transcript models supported by evidence previously annotated in the RefBeet-1.1 assembly [13]. **b** Length distribution of SMRT CCS representing full-length transcripts. **c** Length distribution of transcripts annotated in RefBeet-1.1 that were matched by CCS representing full-length transcripts

We generated large sugar beet cDNA fragments of the reference genotype KWS2320 by using the ‘SMART’ approach [17, 18], which favors the reverse transcription of intact, full-length RNA molecules. In order to equally sample long and short transcripts, the cDNA was size-selected in fractions of lengths 1-2 kb, 2-3 kb, and >3 kb. Using Pacific Biosciences’ SMRT sequencing technology 395,038 cDNA sequencing reads were generated, each consisting of one or more ‘subreads’, which represent the same circularized cDNA template (Fig. 1). A total of 1.1 million subreads were merged into 78,965 circular consensus sequences (CCS), and 626,871 subreads remained unmerged. For 56,546 CCS and 53,374 unmerged subreads we identified the RNA poly(A) tail as well as the SMART cDNA 5' and 3’ primers which are distinct from the PacBio SMRT sequencing adapter. These sequences are referred to as full-insert SMRT reads. Full-length open reading frames (ORFs) could be identified in 98 % of all full-insert SMRT reads by comparison with sugar beet genes that were found to be complete in multiple alignments containing gene sequences from four additional eudicot plant species. The remaining 2 % of cases may be explained by internal priming of short oligo(A) stretches within the coding region. Among the subreads that could not be merged into CCS, there was still a substantial portion of 35.8 % of reads that contained complete ORFs. A general uncertainty remains whether full-ORF sequences also contain a gene’s entire 5' UTR. In line with the expectation that shorter cDNA fragments are more likely to be sequenced full length, the 1-2 kb fraction had the highest percentage of sequences containing both primers (92.2 % of CCS) and the highest percentage of sequences comprising full-length ORFs (94.5 % of CCS, Tables 1 and 2). The length distribution of SMRT read data suggested a genuine representation of expressed sugar beet genes (Fig. 2).

**Table 1:** Proportion of error-corrected SMRT reads containing cDNA primer, poly(A) tail, and canonical polyadenylation signal (AAUAAA). Low levels of the latter are expected, since RNA processing in plants generally shows a decreased dependence on the AAUAAA signal [36]

Dataset	All	With primer (%)	With poly(A) (%)	With primer and poly(A) (%)	With poly(A) signal (%)
CCS 1-2 kb	36,143	92.2	64.3	60.9	21.2
CCS 2-3 kb	20,795	86.4	86.4	79.3	27.3
CCS 3 kb	22,027	86.0	89.1	81.9	29.9
Unmerged subreads 1-2 kb	181,522	13.4	28.5	28.5	9.7
Unmerged subreads 2-3 kb	223,925	10.2	33.5	33.5	11.6
Unmerged subreads 3 kb	221,424	10.6	36.2	36.2	12.6

**Table 2:** SMRT reads covering full ORFs

Dataset	SMRT reads overlapping with full-ORF sugar beet genes^a^	SMRT reads fully covering ORFs (%)	SMRT reads fully covering ORFs and at least 10 UTR bases (%)
CCS 1-2 kb	17,717	94.5	94.2
CCS 2-3 kb	14,846	91.6	91.2
CCS 3 kb	17,706	92.8	92.5
Unmerged subreads 1-2 kb	45,678	41.5	40.7
Unmerged subreads 2-3 kb	70,792	33.6	32.9
Unmerged subreads 3 kb	77,803	34.6	33.9

^a^Interspecific comparison with four other eudicot plants resulted in 7,286 sugar beet genes with *bona fide* complete ORFs

The consensus sequence accuracy could be increased from 97.2 % to 99.0 % for CCS and from 85.2 % to 95.9 % for the unmerged subreads by using the proovread correction software [19] and a normalized dataset of 21 million isogenic sugar beet Illumina mRNA-seq reads generated from public sources [13] (Table 3). Due to the variation in gene expression levels it is not expected to have each gene represented by SMRT sequences. However, high-quality full-length sequencing reads are a valuable resource to establish a reliable training set for gene prediction.

**Table 3:** Accuracy of SMRT transcript sequences before and after error correction using the proovread software

Dataset	Number of sequences	Sequence accuracy	
		Before correction	After correction
CCS	78,965	97.2 %	99.0 %
Full-insert CCS	56,546	97.4 %	98.1 %
Unmerged subreads	626,871	85.2 %	95.9 %
Unmerged full-insert subreads	53,374	86.7 %	94.9 %

**Fig. 3:**
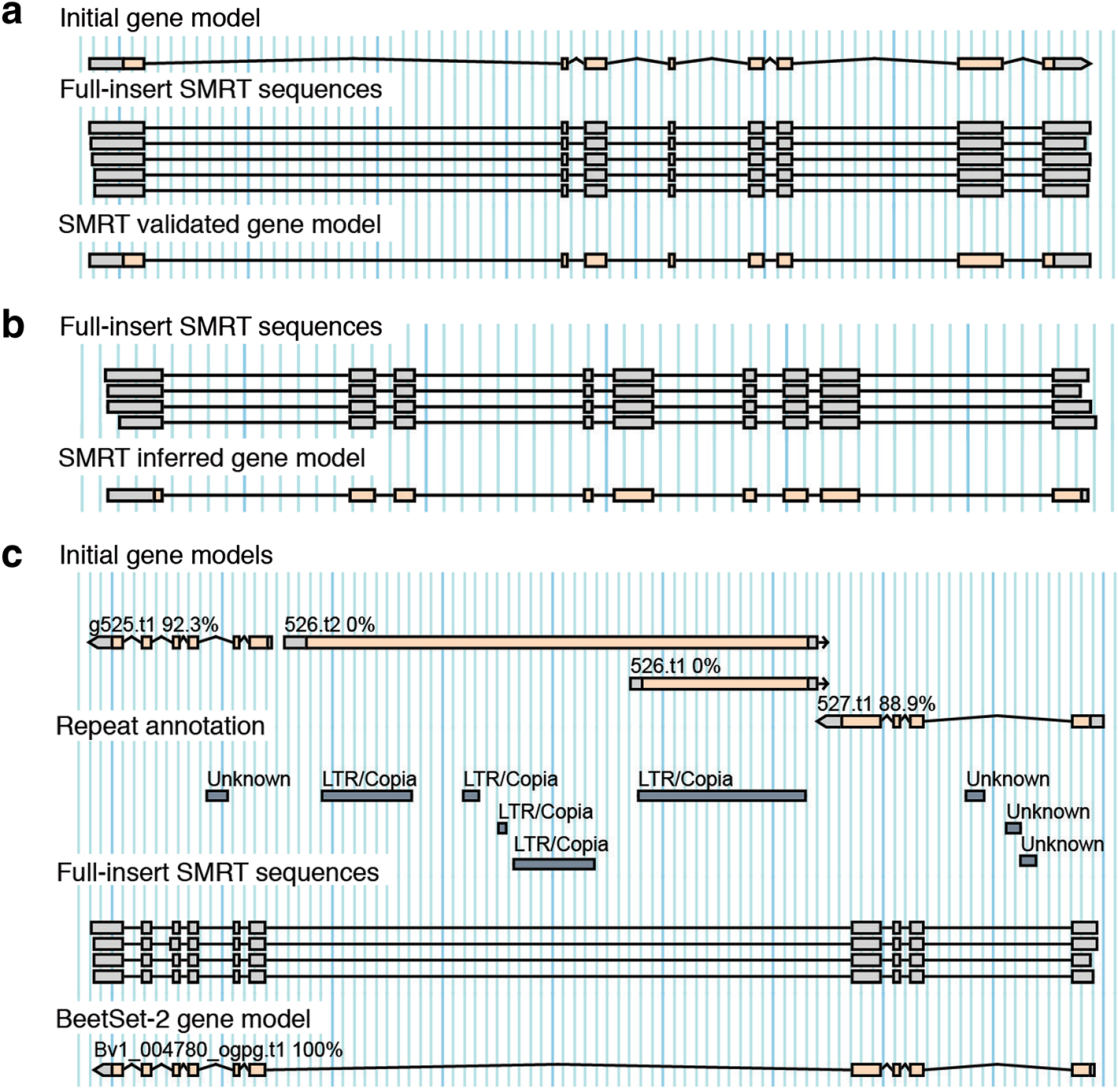
Alignment of full-insert SMRT sequences to identify reliable gene structures. Multiple independent SMRT reads derived from the same gene were used to (**a**) confirm genes previously predicted using AUGUSTUS default parameters and to (**b**) identify new gene models without prior annotation. Gene predictions were considered as validated if all aligning SMRT sequences indicated the same intron boundaries. For new gene models the most abundant isoform per locus supported by at least two reads was reported. **c** Prediction artefact through intronic transposable elements and corrected prediction in BeetSet-2. Numbers next to gene names indicate the percentage of predicted gene features supported by expression evidence

**Fig. 4:**
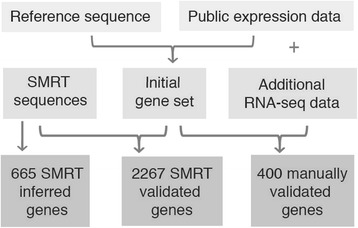
Gene model validation. An initial gene set was calculated based on the sugar beet reference genome (RefBeet-1.2) and publicly available gene expression data [5, 13] using AUGUSTUS default parameters. Genes from the initial gene set were validated using PacBio SMRT sequences and by manual curation. Additional gene models were determined solely from SMRT full-insert sequences. The latter were included to train the parameter set for the final BeetSet-2 gene prediction

By aligning the full-insert SMRT sequencing reads to the sugar beet genome assembly RefBeet-1.2 [13] and by comparing exon coordinates we validated 2,267 gene models that had been generated with the AUGUSTUS software using default *Arabidopsis thaliana* parameters, and accurately predicted 665 additional gene structures solely based on SMRT read alignment (Fig. 3a and b). This step was designed as a time-saving automatic process and did not require manual intervention. An additional 400 genes were manually curated (Fig. 4). The combined set of these reliable gene models was used to select 2,000 non-redundant training genes and 542 test genes to establish optimized AUGUSTUS gene prediction parameters. The specificity of these parameters determine the accuracy of the resulting gene model predictions. To assess the accuracy we calculated the sensitivity and precision separately for UTRs, exons, and the entire transcript, each of which were considered as a ‘feature’ (Table 4). Using a smaller number of genes in the training set resulted in less accurate gene models. However, in smaller training sets SMRT-validated genes performed better than a combination of SMRT-validated and manually validated genes or manually validated genes only (Additional file 1: Table S1), underscoring the robustness of the SMRT-based validation method. Compared to the default *A. thaliana* parameters we obtained improved results after training on *B. vulgaris* genes. However, optimizing *A. thaliana* parameters by *B. vulgaris* genes performed better than generating *B. vulgaris* parameters from scratch. The prediction accuracy remained unchanged when untranslated regions (UTRs) were ignored or when the number of optimization rounds was reduced from 9 to 3.

**Table 4:** Parameter training and results on *ab initio* performance

Training settings		Parameter evaluation on 542 test genes							
Training genes	Setting	Exon level		Transcript level		Sum	Rank	UTR bases	
		Sensitivity	Precision	Sensitivity	Precision			Sensitivity	Precision
288	Manual only	0.759	0.486	0.356	0.201	1.802	9	0.484	0.359
800	-	0.800	0.509	0.424	0.233	1.966	7	0.509	0.350
1,200	-	0.808	0.511	0.445	0.244	2.008	3	0.502	0.358
1,200	New species	0.812	0.491	0.380	0.213	1.900	8	0.480	0.357
1,200	SMRT only	0.820	0.517	0.448	0.245	2.030	2	0.515	0.374
1,200	No UTR	0.808	0.511	0.445	0.244	2.008	3	0.502	0.358
1,200	3 opt. rounds	0.808	0.511	0.445	0.244	2.008	3	0.502	0.358
1,200	6 opt. rounds	0.808	0.511	0.445	0.244	2.008	3	0.502	0.358
2,000	-	0.830	0.515	0.458	0.248	2.051	1	0.508	0.363
*A. thaliana* parameters	-	0.810	0.391	0.384	0.151	1.736	10	0.623	0.287

Manual only: refers to 400 manually curated genes of which 288 were used for training and the remainder as test set. *A. thaliana* parameters: refers to the default *A. thaliana* parameters as provided by Stanke *et al.* Rank: calculated from the sum of the sensitivity and the precision for exons, transcripts, and UTR bases. New species: refers to calculating *B. vulgaris* parameters from scratch using ‘new_species.pl’ (part of the AUGUSTUS pipeline). Opt. round: refers to the number of optimization rounds when running optimize_augustus.pl; the default was nine rounds. SMRT only: refers to training including only SMRT-validated genes. No UTR: refers to not setting the ‘--UTR = on’ parameter when using optimize_augustus.pl

Prediction artifacts include the fragmentation of gene models due to the presence of intronic transposable elements (Fig. 3c) or gene models representing fusions of coding genes with transposon-encoded ORFs. We generated a combined collection of transposable elements by analyzing the genome sequences of sugar beet and spinach [13]. We used the transposable elements either as repeat library for genome masking or as ‘hint’ information during gene prediction and tested the gene prediction accuracy on genomic regions containing gene models derived from SMRT reads. While genome masking initially performed better than repeat hints, we achieved efficient repeat hint usage by increasing their *bonus* factor as predictors of non-exonic regions and by setting their priority level above the priority of expression evidence (Table 5).

**Table 5:** Sensitivity and precision of predicted genes after applying different settings

Setting	Sensitivity in %^a^	Precision in %^b^
Default *A. thaliana* parameter set	71.7	42.4
*B. vulgaris* trained parameter set^c^	82.3	58.9
+ Hint masking	82.9	62.3
+ Hint masking enforcement	83.6	71.8
+ Additional mRNA-seq hints	76.4	44.6
+ mRNA-seq noise reduction	84.7	73.5
+ Higher weighting of introns	85.0	73.9
+ SMRT reads as anchors^d^	91.1	77.9

Settings marked by ‘+’ were added to the setting of the previous line

^a^Percent of correctly predicted transcripts in the set of SMRT derived test genes not overlapping the training gene set

^b^Percent of wrongly predicted genes of all correctly and wrongly predicted gene models in genomic regions of SMRT derived test genes

^c^Training based on SMRT and manually validated genes

^d^‘SMRT reads as anchors’ only affected genes covered by SMRT sequences

**Fig. 5:**
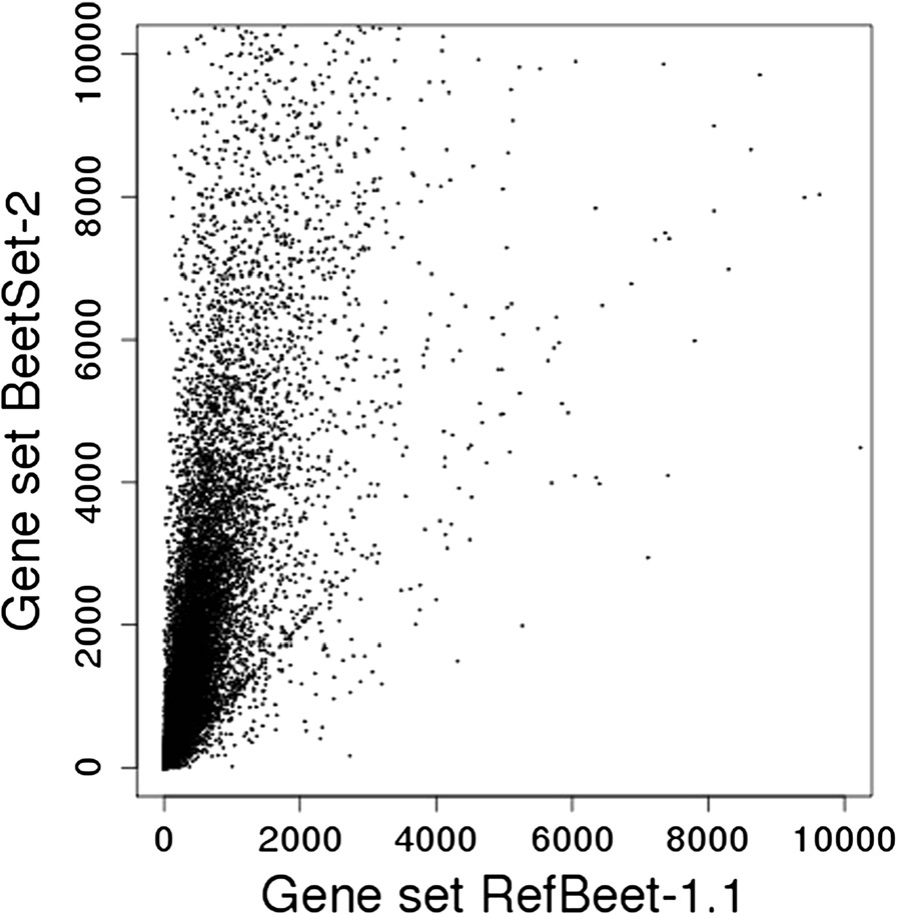
mRNA-seq coverage of sugar beet genes. Each dot represents one sugar beet gene. x-axis: mRNA-seq coverage as in the annotation based on the RefBeet-1.1 assembly; y-axis: mRNA-seq coverage for BeetSet-2 genes. The mRNA-seq data used in the RefBeet-1.1 annotation consisted chiefly of Illumina reads from genotype KWS2320, plus reads from other accessions (total amount: 616.3 million reads). The mRNA-seq data used to generate BeetSet-2 included KWS2320 reads plus isogenic reads from plants grown under stress conditions and their controls (total amount: 923.8 million reads). The overall mRNA-seq coverage increased in BeetSet-2, which improved the prediction of lowly expressed genes

We combined 396.9 million Illumina mRNA-seq reads previously used for sugar beet gene prediction [13] with 526.9 million newly generated reads from sugar beet plants grown under abiotic stress conditions (treated with heat, salt, or high light intensity) and from their untreated controls. All reads were derived from the reference genotype KWS2320. This dataset of almost 1 billion quality-filtered mRNA-seq reads (Table 6) led to increased evidence levels for a large number of genes with low or intermediate level of expression: 8,201 genes with average mRNA-seq read coverage below 200x in the published gene set [13] increased their coverage by at least two-fold (Fig. 5). However, adding more expression evidence resulted in higher level of background noise, due to interference of, for example, rare isoforms or incompletely spliced mRNAs, which affected the prediction accuracy (Table 5). We reduced the noise by applying coverage filters (see Methods for details) to facilitate the correct prediction of the most abundant isoform per locus, being aware that in this way low abundance isoforms might be lost. The noise reduction improved the sensitivity from 76.4 % to 84.7 %. We further increased the *bonus* factor for intron hints, and increased the *malus* factor for predictions that did not coincide with intron hints. In combination with these improved settings repeat hint masking performed slightly better than genome masking. Using pre-assembled mRNA-seq reads as additional EST hints did not increase the sensitivity. SMRT full-insert sequences as additional EST hints only slightly improved the prediction result, due to the shallow coverage of such reads. Increasing their weight by conversion to ‘anchors’ increased the sensitivity to 91 %.

**Table 6:** Expression support of sugar beet genes

Source	Input sequences	Supported genes
ESTs	35,523	10,222
Roche/454 sequences	282,169	12,681
SMRT full-insert	109,793	3,874
KWS2320 mRNA-seq (all reads)	923.8 million	26,369
KWS2320 salt stress	86.2 million	21,974
KWS2320 heat stress	91.6 million	22,166
KWS2320 light stress	130.0 million	23,041
Sum	924.2 million	26,409

Supported genes: genes partially or completely supported by expression evidence

In this work, we benchmarked the various settings in Caryophyllales species. However, the main observations are expected to be profitable for gene predictions in other clades and species. This includes improvements regarding: the number of training genes, the masking procedure, additional mRNA-seq hints, mRNA-seq noise reduction, higher weight for introns, training optimization rounds, and SMRT reads as anchors.

Taking advantage of all optimized settings and using 2,794 non-redundant validated genes as training set, we predicted an improved genome-wide gene set for sugar beet (Table 7). The final gene set is referred to as BeetSet-2 and consists of 26,923 genes (Table 8).

**Table 7:** Final parameter training using 2,794 training genes and results on *ab initio* performance

	Parameter evaluation on 349 test genes							
Training parameter	Exon level		Transcript level		Sum	Rank	UTR bases	
	Sensitivity	Precision	Sensitivity	Precision			Sensitivity	Precision
*A. thaliana* parameters	0.810	0.527	0.368	0.192	1.899	2	0.678	0.281
*B. vulgaris* parameters	0.842	0.664	0.461	0.341	2.308	1	0.547	0.508

**Table 8:** Number of predicted genes in sugar beet and spinach

	Gene sets sugar beet		Gene set spinach
	Annotation in RefBeet-1.1	BeetSet-2	SpiSet-1
100 % evidence	16,508	17,434	12,664
1-99 % evidence	15,556	8,975	7,868
0 % evidence	80,439	13,181	19,777
0 % evidence and 1:1 orthology^a^	n.a.	514	1,171
Final gene number	27,421^b^	26,923^c^	21,703^c^

^a^One-to-one orthology identified between BeetSet-2 and SpiSet-1 predictions

^b^Genes annotated in RefBeet-1.1 with 1-100 % expression evidence excluding those with transposable element homology

^c^Sum of genes with 1-100 % expression support and one-to-one orthologs

The number of sugar beet genes located in chromosomally assigned scaffolds increased by 10.4 % to 84.0 %, mainly due to the improved continuity of the employed reference assembly RefBeet-1.2. These genes have an average gene length of 5,887 bp including introns, an average coding sequence length of 1,134 bp and 4.8 coding exons per gene. Stable gene identifiers (IDs) of the previous sugar beet gene annotation [13] could be transferred to 88.2 % BeetSet-2 genes, and unique IDs were assigned to the remaining 3,164 genes. The percentage of genes with more than one predicted isoform decreased slightly from 6.8 % to 6.7 %.

The full-insert SMRT reads allowed us to assess the variability of UTR lengths. The median of the 3' UTR length, measured as the distance between the translational stop codon and the first base of the poly(A) tail, was 242 bp with a median variation of 59 bases. The median length of 5' UTRs (distance between read start and translation start site) was 104 bases with a median variation of 30 bases.

Based on manual inspection of 200 randomly selected sugar beet genes we estimated that genome-wide about 4,000 genes had been incorrectly predicted in the previous version but are correctly annotated in BeetSet-2, which is consistent with the improved sensitivity (Table 5). The number of genes with 100 % expression evidence increased by 5 % whereas the number of genes with 1-99 % evidence decreased by 42 %. A total of 3,874 genes were covered by expression evidence derived from full-insert SMRT-reads (Table 6), and 26,369 genes were covered by mRNA-seq reads, demonstrating the complementary role of long-read and short-read data in evidence-based gene prediction. Further evidence genes had support from SMRT reads that represented parts of transcripts. However, applying them as additional hints did not improve the overall prediction accuracy (data not shown). Although mRNA-seq reads from plants grown under stress conditions increased the level of expression evidence for many genes, there were only three genes in BeetSet-2 that were exclusively covered by stress-condition data.

We applied the *B. vulgaris* parameters to predict a genome-wide gene set in the spinach genome [13], a close relative of sugar beet. Public sequence databases at the time of this study contained only 561 EST and mRNA sequences from spinach and included redundant entries. Using 108.0 million mRNA-seq reads from an inbred spinach Viroflay genotype, we predicted 20,532 spinach genes supported by expression evidence and 19,777 spinach genes without expression evidence. Since genes are expected to be conserved between related species, sequence homology of coding regions in sugar beet and spinach was considered as confirmation of the predicted gene models. Based on a blast reciprocal best hit approach [20], 14,735 orthologous genes (1:1 orthology relationship) between sugar beet and spinach were detected. All genes with expression evidence as well as 514 sugar beet genes and 1,171 spinach genes currently without expression evidence but with 1:1 orthology were included in the final gene sets. In spinach, we performed gene predictions both with AUGUSTUS’ default *A. thaliana* parameters and with *B. vulgaris* trained parameters. The number of correct gene models increased by 6 % when using *B. vulgaris* parameters as assessed on 200 randomly selected spinach genes. Of genes with partial expression evidence we found 17 % more spinach gene models correctly predicted.

## Conclusions

Like the spinach genome, yet unexplored taxa are now in immediate reach for molecular characterization due to the drastic decrease of per-base sequencing costs over the last years. Each of these new genomes may have evolved in their own specific way so that gene annotation needs to be adapted to their properties or to closely related species. Here, we have benchmarked various steps in the *de novo* annotation of coding sequences. Our results show that PacBio SMRT full-length cDNA sequences are well suited to identify reliable gene models and to fine-tune prediction parameters. We developed the SMRT-based validation as an automated process to overcome time-consuming manual curation. This pilot work is based on PacBio C2 sequencing chemistry and XL polymerase. However, more advanced PacBio chemistries have meanwhile been released, increasing the average read length and the overall run output. It is thus conceivable that the strategy outlined herein will be even more successful using newer sequencing chemistry. By applying adjusted filtering settings optimal advantage can be taken from deep short-read transcriptome sequencing, and providing locations of transposable elements can reduce prediction artifacts. The improved parameters were used for calling genes in sugar beet and in its sister taxon spinach, two plants quite distinct from most other sequenced taxa. Predicted genes that currently lack transcription evidence were verified through the identification of 1:1 orthologues in sugar beet and spinach. The workflows described will be of importance to explore the genomes of lesser known eukaryotes, and the new gene sets for sugar beet and spinach are valuable resources for plant research and comparative genomics.

## Methods

### Genome assemblies

Genes were predicted based on sugar beet assembly RefBeet-1.2 [13] and spinach assembly Spinach-1.0.1 (assemblies accessible at [21]). Adapted assembly versions compliant with GenBank submission specifications can be accessed at GenBank with accession numbers AYZS02000000 (RefBeet), and AYZV02000000 (spinach).

### Sample preparation and SMRT sequencing

Sugar beet seedlings were obtained by incubation of seeds of sugar beet DH line KWS2320 at 20 °C for 48 h. Seedlings were removed from the dish once cotyledons had become fully visible. Seeds that had not germinated were discarded; the success of germination was about 75 %. Total RNA was isolated from the seeds using the Nucleospin Plant RNA kit (Macherey-Nagel, Düren, Germany). Ten nanograms of total RNA was reverse transcribed using the SMARTer PCR cDNA Synthesis Kit (Clontech, Mountain View, CA, USA), and cDNA was amplified using the Advantage 2 PCR kit (Clontech) for 12 cycles. The generated cDNA was then re-amplified in nine independent PCR reactions using the Advantage 2 PCR kit and the IS primer (0.4 μM final concentration) for 30 cycles. A total of 1 μl of generated cDNA was used in each reaction. Re-amplified cDNA was purified using the QIAquick PCR Purification kit (Qiagen, Hilden, Germany) and thereafter size-selected on agarose gels into cDNA fractions of length 1-2 kb, 2-3 kb, and >3 kb. Excised fractions were column-purified using the QIAquick Gel Extraction kit (Qiagen). The fragment size distribution was validated on a Bioanalyzer HS chip (Agilent, Santa Clara, CA, USA) and quantified on a Qubit fluorometer (Life Technologies, Carlsbad, CA, USA). The three cDNA size fractions were submitted to KeyGene N.V. (Wageningen, the Netherlands), where library preparation and SMRT sequencing on the Pacific Biosciences RS sequencing instrument was carried out. MacBead loading and SMRT C2 sequencing chemistry were used together with XL polymerase. Two SMRT cells were run from each of the three fractions, that is, one movie each of 1 × 120 min and one movie each of 2 × 55 min.

### Data analysis overview

**Fig. 6:**
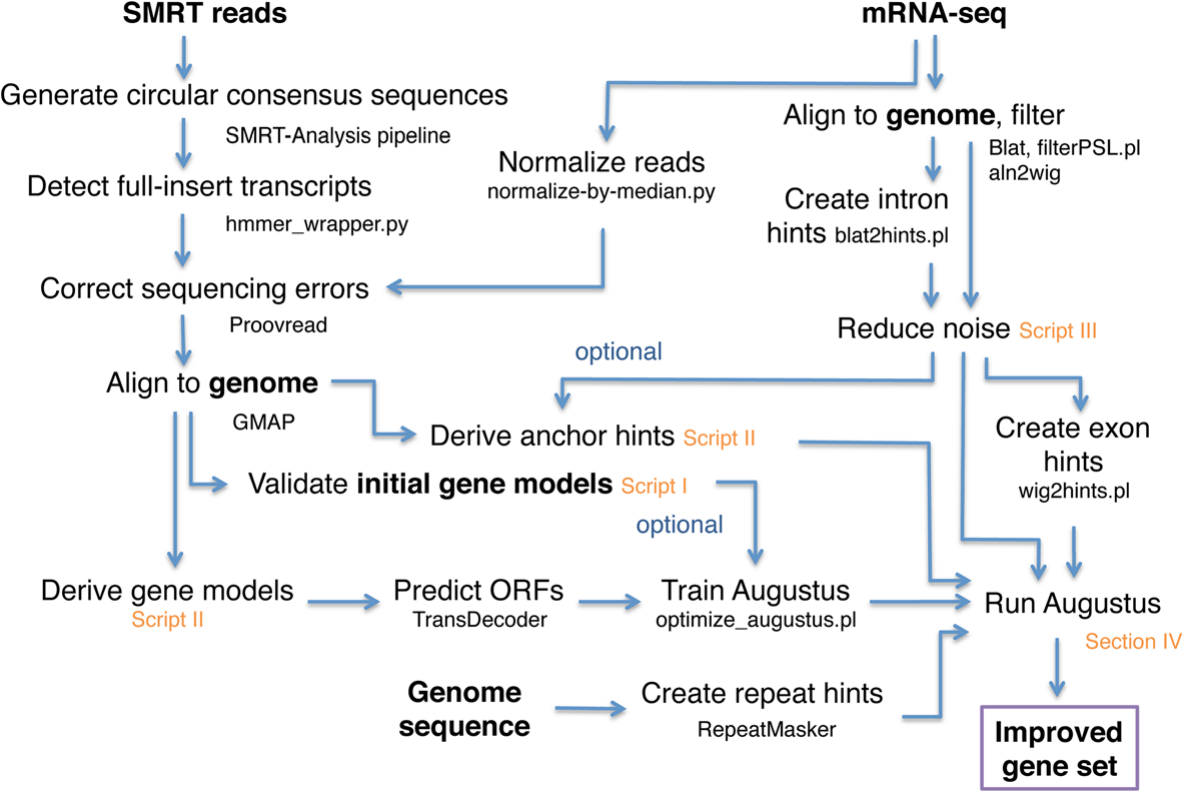
Workflow of our analyses to improve eukaryotic gene predictions, including the scripts that are part of this publication (highlighted orange). Input and output data are highlighted in bold lettering

The analysis steps are summarized in Fig. 6.

### Pre-processing of SMRT reads

Raw SMRT sequencing reads consist of one or several subreads representing the same circularized cDNA template separated by adapter sequence. Subreads smaller than 50 bp and reads with a quality less than 0.75 (corresponding to a predicted error rate of >25%) were removed. Remaining subreads were merged into consensus sequence (CCS) reads, whenever the entire cDNA template was covered by at least two subreads, using the Pacific Biosciences SMRT-Analysis pipeline v2.0. Full-insert sequencing reads were identified from single-pass subreads and CCS reads by running hmmer_wrapper.py [22]. Reads with both 5' and 3' primer sequences and a poly(A) tail present were considered to represent full-length transcripts. Primer sequences as well as the poly(A) tail were trimmed off prior to further analysis.

### Identification of SMRT reads containing full-length ORFs

The percentage of SMRT reads containing complete ORFs was estimated from SMRT reads overlapping full-ORF BeetSet-2 genes. SMRT CCS and unmerged subreads were aligned to the reference sequence RefBeet-1.2 with GMAP (v2012-07-20, -A -f gff3_match_cdna -B 5). The CDS of 9,422 BeetSet-2 genes was covered (> = 1 base overlap) by one or more SMRT reads. For the BeetSet genes only the transcript with the longest CDS was considered. The completeness of BeetSet-2 genes was inferred from multiple protein alignments to four different eudicot plant species. Protein sequences were downloaded from plants.ensembl.org: *A. thaliana* (TAIR10.24), *V. vinifera* (IGGP_12x.24), *S. tuberosum* (3.0.24), *P. trichocarpa* (JGI2.0.24). The corresponding protein sequences of the 9,422 BeetSet-2 genes were aligned to the protein sequence of these four other plant species using BLAT (v34, proteins, default). Alignments with less than 50 % matching bases of BeetSet-2 proteins were discarded. Each BeetSet-2 gene and the most similar gene from the other species (at most one per species) were realigned in a multiple alignment step using ClustalW (v1.83). BeetSet-2 genes were regarded as complete if they had either the same start and end coordinates or additional amino acids compared to at least one other species. A total of 7,286 BeetSet-2 genes passed these criteria. 244,542 PacBio reads partially or fully overlapped with these BeetSet genes. The percentage of SMRT reads with full length ORF was calculated from this subset. A SMRT sequence was considered full ORF, when the entire ORF of the underlying sugar beet gene was included in the SMRT sequence alignment. UTR was considered as present if at least 10 additional bases up- and downstream of the BeetSet-2 ORF were present.

### Correction of SMRT sequences

Full-insert transcripts determined from CSS and unmerged subreads, remaining CCS, and remaining unmerged subreads were corrected separately with proovread [19] (default settings) using quality-filtered and trimmed Illumina reads [13, 23]. The Illumina data used for correction had been generated from inflorescence tissue of the sugar beet reference genotype KWS2320. In order to accelerate SMRT data correction, Illumina reads were normalized to a maximum coverage of 100-fold per transcript using normalize-by-median.py (parameters: -N 4 -x 48e9 -k 17 -C 100) of the khmer package [24]. Normalization excluded 70 % of the Illumina reads, leaving 21,132,501 reads for the correction. By default, proovread performs quality trimming after error correction, but also provides the error-corrected untrimmed reads. To maintain the full length of the processed SMRT sequences only the corrected but untrimmed SMRT sequences were used for further analysis.

To determine the sequence accuracy of the sugar beet SMRT data before and after error correction, the sequences were aligned against the set of protein coding gene transcripts (v1302) predicted in the sugar beet assembly RefBeet-1.1 using blasr [25, 26] (part of SMRT analysis pipeline v2.0, assembly and gene set available on [21]). For each SMRT sequence only the best alignment was retained and only if at least 90 % of its length matched. The accuracy was determined based on the average alignment identity. RefBeet-1.1 gene predictions have high consensus accuracy [13]. Uncertainties in the accuracy estimation due to potential structural errors in the previously predicted transcripts were avoided by only considering full-length aligning SMRT reads (>= 90% of length).

### Stress conditions and mRNA-seq data

KWS2320 sugar beet plants for abiotic stress treatment (salt, heat, light, and control) were grown in hydroculture in Hoagland’s medium with weekly exchange under a 10 h light/14 h dark cycle at constant temperature of 21 °C, 60 % relative humidity, and light intensity of 80–120 μmol m^−2^ s^−1^. For the salt treatment, the plants were transferred into fresh nutrient solution with 50 mM NaCl at day 23 after sowing. The salt concentration was stepwise increased to 300 mM at day 28 and then kept until harvesting. For the light treatment plants were exposed to a light intensity of 800 μmol m^−2^ s^−1^ for 4 h prior to harvest. The heat treatment was performed by exposing the plants to a temperature of 35 °C for 3 h prior to harvest. All plants were harvested 30 days after germination, and the material was immediately frozen in liquid nitrogen. Total RNA was isolated from leaves by phenol:chloroform:isoamylalcohol (25:24:1) extraction and LiCl precipitation. The resulting RNA was treated with DNA-free DNAse (Life Technologies), and subsequently quantified. Success of the stress treatment was validated by detection of differential gene expression for genes expected to be stress-responsive. Among the abiotic stress-induced genes were MYB12, CHS (encoding chalcone synthase), ELIP genes encoding early light-inducible proteins, as well as genes coding for ethylene-responsive transcription factor, heat shock proteins and glutathione S-transferases. Among the 120 genes which showed the highest differential expression (>100-fold) between stressed conditions vs. controls were five genes previously predicted in RefBeet-1.1 with an expression evidence of <1 %.

Non-directional cDNA libraries were sequenced on a HiSeq1500 instrument (Illumina, San Diego, CA, USA) according to the instructions of the manufacturer. Spinach mRNA-seq was performed on an inbred Viroflay variety (Syngenta Seeds, the Netherlands), isogenic to the published reference assembly Spinach-1.0.1 [13]. RNA was isolated from young leaves and apical shoots using a Nucleospin RNA Plant kit (Macherey-Nagel, Düren, Germany). Non-directional mRNA-seq libraries were prepared using Illumina kit RS-100-0801, and were sequenced on the Illumina HiSeq2000 using a 2 × 50 cycle paired-end sequencing protocol.

### Identification of accurate sugar beet gene models for parameter training

An initial gene set was predicted in the sugar beet reference assembly RefBeet-1.2 with AUGUSTUS v2.7 using the same settings and expression data (mRNA-seq data: SRA accessions SRX287608-SRX287625, Roche/454 data: SRA accession SRX287606, 35,523 ESTs) as in the RefBeet-1.1 annotation v1302 [13]. Error-corrected SMRT sequences were mapped to RefBeet-1.2 using Gmap [27], parameters -A -f gff3_match_cdna -B 5. SMRT sequences aligning to multiple locations or not mapping at their full length were removed. To account for deletion errors, SMRT sequences were considered to map at full length if they aligned with at least 90 % of their lengths, missing at most 50 bases. Gene models of the initial gene set in gff format were validated by SMRT sequences using the custom Perl (v5.10.1) script ‘validate-gene-models-with-PacBio.pl’ (Additional files 2, 3, and 4). A gene model was considered validated if all SMRT sequences aligning to the exons of the gene model confirmed the same number and order of exons as well as exactly the same intron boundaries. The UTR length was allowed to be variable, predicted start and stop codons were required to be covered. Thirty-two genes at scaffold borders (within the first or last 5,000 bases) were removed, since they may represent incomplete genes. In total, 2,267 SMRT gene models were validated by this method.

Due to sequencing errors in SMRT sequences, intron boundaries of the initial gene models did not always coincide with those of the aligned SMRT sequences. For such cases and for cases in which the initial gene model had been erroneously predicted, additional gene models were derived directly from aligned SMRT sequences. Gene models were clustered based on their alignment location and intron boundaries using the custom Perl script ‘derive-gene-models-from-PacBio.pl’ (Additional files 2, 3, and 4). The most abundant isoform per location was selected. Transcript boundaries were derived from the median start and stop positions of all aligned SMRT-sequences representing a selected isoform. Open reading frames (ORFs) were predicted with TransDecoder (Brian Haas *et al.*, unpublished [28]). In this way, 665 additional gene models, non-overlapping with previous SMRT-validated genes, were obtained. The SMRT-based validation was developed as a fast and entirely automatic process without the need of manual curation.

For the manually validated genes, arbitrarily chosen RefBeet-1.1 gene predictions with a hint coverage of at least 85 % were extracted using a customized Perl script ‘parse_AUGUSTUS_gff3.pl’ (Additional files 2, 3, and 4). The exon number of candidate genes was defined based on the intron-exon distribution of the RefBeet-1.1 gene set. Genes were manually inspected using GenomeView [29], visualizing RefBeet-1.1 gene predictions and supporting mRNA-seq evidence. Gene structures were manually inspected and corrected where necessary. In total, 400 validated gene structures were obtained after manual curation.

### Training and testing gene prediction parameters

Gene prediction parameter sets specific to *Beta vulgaris* were trained by the supervised machine learning algorithm implemented in the AUGUSTUS pipeline; we followed the general guidelines indicated in [30]. To select training genes and test genes we combined the set of SMRT-validated and manually validated genes based on initial AUGUSTUS predictions (the 665 SMRT-derived genes were not included at this stage). GenBank files containing the genes together with flanking intergenic regions were generated with gff2gbSmallDNA.pl. As detailed in the instructions on how to train AUGUSTUS, redundant genes and genes with a CDS length not divisible by 3 were removed. A gene was considered redundant if it had a sequence identity of 80 % or larger in an all-versus-all blat [31] protein sequence alignment. The remaining total of 2,542 validated genes were randomly subdivided into 2,000 training genes of which different subsets were used (Table 2) and 542 test genes for the calculation of sensitivity and precision using AUGUSTUS. In two separate approaches we: (1) trained parameters from scratch using the new_species.pl script; and (2) optimized existing *A. thaliana* parameters included in the AUGUSTUS package using the optimize_AUGUSTUS.pl script. The sensitivity and precision were calculated separately for UTRs, exons, and the entire transcript (referred to as ‘features’). The sensitivity was calculated by dividing the number of correctly predicted features (true positives) by all features in the test gene set (true positives and false negatives) and the precision by dividing the number of correctly predicted features (true positives) by all predicted features within the genomic regions of the test gene set (true positives and false positives).

### Masking transposable elements

Repeats were identified and classified using the RepeatModeler software [32], v1.0.7, downloaded from [33]) applied on the sugar beet assembly RefBeet-1.2 and the spinach assembly Spinach-1.0.1. Repeat sequences classified as transposable elements were selected in both species (searching for ‘DNA, LTR, LINE, SINE, or Helitron’ in the RepeatModeler output). The repeat catalog of the sugar beet assembly RefBeet-1.1 had been manually curated [13], resulting in a number of repeats identified as transposons or retrotransposons which were ‘Unknown’ or ‘Simple repeat’ according to RepeatModeler. Those sequences were included in the combined sugar beet and spinach collection of transposable elements. The collection was used as input for RepeatMasker (further parameters: -gff -no_is -norna -nolow) to mask the sugar beet assembly RefBeet-1.2. Positions of transposable elements in RefBeet-1.2 were converted into repeat hint annotation interpretable by AUGUSTUS (source = RM in gff hint file). In one approach, genes were predicted on the repeat-masked genome, and in a second approach, the unmasked genome was used along with the positions as repeat hint information. To enforce the exclusion of repeat regions from gene models, the *bonus* factor for the prediction of repeat hints as non-exonic regions was increased from 1.01 to 1e + 10, and the repeat hint priority level was set to 6 (priority=6 in gff), which is above the default priority level of expression evidence of 4. The effect on the gene prediction accuracy of both masking approaches was evaluated by applying AUGUSTUS on genomic regions containing SMRT-derived genes (GenBank format) and by manual inspection of 200 genes of the genome-wide prediction in sugar beet (see ‘Manual quality assessment of gene predictions’). Both evaluations showed a slightly higher number of correctly predicted genes for hint masking when combined with additional expression hints and higher intron hint weighting (sensitivity hint masking 85.0 % versus sequencing masking 84.5 %). For gene annotation in Spinach-1.0.1, transposons were only provided to AUGUSTUS as repeat hint annotation.

### Processing of expression evidence

KWS2320 sugar beet mRNA-seq data from stress conditions and their controls were quality-filtered and trimmed according to criteria described elsewhere [23], resulting in 526.9 million reads. mRNA-seq data that had been employed in predicting RefBeet-1.1 genes consisted of 616.3 million reads of which 396.9 were derived from genotype KWS2320. For this work, only mRNA-seq data of the KWS2320 genotype were utilized, in total 923.8 million quality-filtered reads. The reads were aligned to RefBeet-1.2 with blat, and processed and converted into hint information for AUGUSTUS as described previously [13].

mRNA-seq expression data were filtered using the custom Perl script ‘RNA-seq-noise-reduction.pl’ (Additional files 2, 3, and 4) to reduce the background noise and to facilitate the prediction of the most abundant isoform per locus correctly taking into account that some isoforms were not reported. The mRNA-seq coverage was reduced by 10 % of the local peak coverage (95 percentile, in 1-kbp windows). If the coverage difference between two overlapping intron hints (gff format) was greater than 90 % the intron hint with the lower coverage was removed. The mRNA-seq coverage (wig format) was reduced within boundaries of introns by 50 % of the adjacent exon coverage. Introns were considered if they were smaller or equal than 50 kbp in size and showed a coverage drop of at least 50 % at their exon-intron junction when comparing the coverage 10 bases upstream and downstream of each junction. To increase the weight of intron hints derived from mRNA-seq data (*bonus* factor 1), anchors were added for introns that were supported by at least 50 aligned mRNA-seq reads (source = M, *bonus* factor 1e + 100). The intron *malus* factor was adjusted from 0.34 to 0.001.

Aligned full-insert SMRT sequences (gff format) were converted into hints using the custom Perl script ‘derive-gene-models-from-PacBio.pl’ (Additional files 2, 3, and 4). In brief SMRT sequences were clustered based on their location and intron boundaries. Per location, the most abundant isoform was converted to exon hints, exon part hints (for terminal exons) and intron part hints, grouped together by using the group tag (gff column 9). The source tag of these hints was set to E when employed as EST hints and to M when employed as anchors. It was required that an isoform was represented by at least two SMRT sequences or by one SMRT sequence for which intron boundaries were confirmed by aligned mRNA-seq reads. The terminal exon positions were set to the median alignment start and stop positions of all SMRT sequences representing a selected isoform. In order to prevent AUGUSTUS from appending exons to SMRT hint groups, flanking intergenic hints of length one were added at a distance of 50 bases.

### Calculation of an optimized sugar beet gene set

For the final parameter training, SMRT-validated genes, SMRT-derived genes, and manually validated genes were combined. In order to exclude additional genes from the flanking regions of the training genes, non-overlapping genes of the initial prediction were temporarily added to generate the GenBank file using gff2gbSmallDNA.pl. Redundant and problematic genes were removed as described above, and 2,794 training genes and 349 test genes were randomly selected using randomSplit.pl. Parameters were trained with optimized settings as evaluated above and using optimize_AUGUSTUS.pl. The extended training gene set resulted in further improvement of the *ab initio* performance (Table 4 and 7). The sugar beet reference gene set ‘BeetSet-2’ was generated on the assembly RefBeet-1.2 using AUGUSTUS version 2.7 with the newly generated *B. vulgaris* parameters, filtered sugar beet KWS2320 mRNA-seq hints, KWS2320 SMRT hints as well as Sanger and Roche/454 EST hints (Table 4; Additional files 2, 3, and 4). The options to predict and print UTRs were switched on, the gene model was set to ‘complete’ and no in-frame stop codons were allowed.

### Transferring stable sugar beet gene identifiers

From the previous sugar beet gene set [13], the longest CDS per gene was mapped against RefBeet-1.2 using gmap (version 2012-07-20, -A -f gff3_match_cdna -B 5 -t 20). A stable identifier of a previously annotated gene was transferred to the BeetSet-2 gene with the longest summed CDS match length using the custom Perl script ‘transfer-stable-stable-identifier.pl’ (Additional files 2, 3, and 4). Whenever the CDS overlap was equally long to multiple genes, the gene order was tried to be preserved. Considering both evidence and non-evidence genes, in 558 cases one previously annotated gene overlapped multiple adjacent BeetSet-2 genes. In these cases the identifier was transferred to the BeetSet-2 gene with the longest partial CDS overlap. In 2,020 cases, multiple adjacent previously annotated genes matched best to a single BeetSet-2 gene. In these cases the identifier of the best reciprocal matching previously annotated gene was transferred. New stable identifiers were assigned to the remaining BeetSet-2 genes.

### Manual quality assessment of gene predictions

Of the previous sugar beet gene set calculated with default *A. thaliana* parameters [13], 100 genes with 100 % evidence and 100 genes with 1-99 % evidence were randomly selected. The evidence for a gene was considered to be 100 % if all CDS, UTRs, and introns were supported by expression data. Genes and expression evidence were visualized in Gbrowse [34], and the correct structure was inferred from the combined Illumina mRNA-seq, SMRT, Sanger, and Roche/454 expression data. The number of correct structures was compared between the annotation in RefBeet-1.1 and BeetSet-2. Spinach genes predicted with *A. thaliana* or *B. vulgaris* parameters were inspected in the same way, except that genes at scaffold borders were kept.

### Calculating number of stress-condition specific genes

Genes specifically supported by mRNA-seq reads from plants under stress conditions were determined by selecting those genes with no expression hint coverage under non-stress conditions and at least 90 % coverage of the CDS length under stress conditions (salt, heat, or light).

### Applying *Beta vulgaris* parameters on spinach gene prediction

Newly generated *B. vulgaris* parameters were used together with 108.0 million quality filtered spinach mRNA-seq reads (RNA isolated from spinach leaves) to predict genes in the Spinach-1.0.1 genome assembly. Hint information was produced from these mRNA-seq data (isogenic to assembly) and processed in the same way as sugar beet mRNA-seq data.

### Prediction of 1:1 orthologous genes between sugar beet and spinach

Evidence and non-evidence genes were combined. Per gene, the transcript encoding the longest protein was selected. Protein sequences of spinach and sugar beet were aligned to each other using blastp [35] (expect cut-off 1e-5, minimum alignment length 50 % of protein length). 1:1 orthologous genes were predicted using the reciprocal best blast hit approach [20].

### Data access

Raw transcript sequencing data from sugar beet genotype KWS2320 and spinach genotype Viroflay were deposited in the NCBI Sequence Read Archive (SRA) with the following accession numbers: SRX674050 (sugar beet Pacific Biosciences SMRT sequences); SRR1508751, SRR1508753, SRR1508755, SRR1508756, and SRR1508758 (Illumina sugar beet mRNA-seq data from stressed and unstressed plants); SRX674044 (spinach mRNA-seq). Sugar beet and spinach gene models, assemblies, sugar beet SMRT validated genes used for training corrected SMRT sequences, and hints and anchors for Augustus gene prediction can be downloaded from [21]. This site further includes a genome browser for visualizing sugar beet and spinach annotation. Gene models were deposited on GenBank and have been assigned stable locus identifiers BVRB_1g000010 - BVRB_1g023380, BVRB_2g023390 - BVRB_2g047930, BVRB_3g047940 - BVRB_3g070810, BVRB_4g070820 - BVRB_4g097640, BVRB_5g097650 - BVRB_5g127170, BVRB_6g127180 - BVRB_6g156500, BVRB_7g156510 - BVRB_7g180820, BVRB_8g180830 - BVRB_8g202200, BVRB_9g202210 - BVRB_9g226140, and BVRB_000010 - BVRB_043090 for sugar beet, and SOVF_000010 - SOVF_217030 for spinach. Genome assemblies have been assigned accession numbers AYZV02000000 (spinach) and AYZS02000000 (sugar beet). GenBank gene sets and assemblies were adapted to be compliant with GenBank submission specifications.

## Additional files

Additional file 1: Table S1. Gene model parameter training. (DOC 30 kb) Additional file 2:
**Description of scripts for generation and validation of gene models, RNA-seq noise reduction, and other custom-written perl scripts.** (DOCX 36 kb) Additional file 3:
**Perl scripts.** (ZIP 19 kb) Additional file 4:
**Sample data for testing pipeline functionality.** (ZIP 18664 kb)

## Abbreviations

CCS: Circular consensus sequence
EST: Expressed sequence tag
ID: Gene identifier
SMRT: Single-molecule real-time
UTR: Untranslated region
