# Supplementary material for: Exploiting single-molecule transcript sequencing for eukaryotic gene prediction
Source: Genome Biol. 2015 Sep 2;16(1):184. doi: 10.1186/s13059-015-0729-7 (PMC4556409; doi:10.1186/s13059-015-0729-7)
Supplement: Additional file 1: Table S1. — Gene model parameter training. (DOC 30 kb) [file 13059_2015_729_MOESM1_ESM.doc]

**Additional file 1: Table S1**: Gene model parameter training

| Parameter set | Exon | | Transcripts | | UTR bases | |
| --- | --- | --- | --- | --- | --- | --- |
|  | Sensitivity | Precision | Sensitivity | Precision | Sensitivity | Precision |
| *A. thaliana* | 0.812 | 0.527 | 0.368 | 0.192 | 0.678 | 0.280 |
| *B. vulgaris* 400 manually validated | 0.820 | 0.625 | 0.406 | 0.286 | 0.56 | 0.352 |
| *B. vulgaris* SMRT + manually validated | 0.861 | 0.687 | 0.472 | 0.334 | 0.597 | 0.382 |

Tested on genomic regions containing gene models derived solely from SMRT sequences; these genes were not used for training
