# Supplementary material for: Exploiting single-molecule transcript sequencing for eukaryotic gene prediction
Source: Genome Biol. 2015 Sep 2;16(1):184. doi: 10.1186/s13059-015-0729-7 (PMC4556409; doi:10.1186/s13059-015-0729-7)
Supplement: Additional file 2: — Description of scripts for generation and validation of gene models, RNA-seq noise reduction, and other custom-written perl scripts. (DOCX 36 kb) [file 13059_2015_729_MOESM2_ESM.docx]

**Exploiting single-molecule transcript sequencing for eukaryotic gene prediction**

André E. Minoche^1,2,3^, Juliane C. Dohm^1,2,3,4^, Jessica Schneider^5^, Daniela Holtgräwe^5^, Prisca Viehöver^5^, Magda Montfort^2,3^, Thomas Rosleff Sörensen^5^, Bernd Weisshaar^5,*^, Heinz Himmelbauer^1,2,3,4,*^

^1^Max Planck Institute for Molecular Genetics, Berlin, Germany; ^2^Centre for Genomic Regulation (CRG), Barcelona, Spain; ^3^Universitat Pompeu Fabra (UPF), Barcelona, Spain; ^4^University of Natural Resources and Life Sciences (BOKU), Vienna, Austria; ^5^Department of Biology/Center for Biotechnology, Bielefeld University, Bielefeld, Germany

*Corresponding authors:

Heinz Himmelbauer, E-mail: heinz.himmelbauer@boku.ac.at

Bernd Weisshaar, E-mail: bernd.weisshaar@ uni-bielefeld.de

**SUPPLEMENTARY DATA 1**

## I. Validation of Augustus gene models using SMRT reads from the Pacific Biosciences (PacBio) platform

Augustus gene predictions can be improved by training on species- or clade-specific gene models. If a species has no or too few confirmed gene models, more gene models can be created from an initial round of gene predictions using prediction parameters of a related species plus expression evidence (e.g. mRNA-seq data). For training only high-confidence gene models should be used. The provided script validates initial gene models using aligned full-insert error-corrected PacBio reads.

perl scripts/validate-gene-models-with-PacBio.pl PacBio-validation data/sample.initial-gene-models.gff data/sample.aligned-PacBio-reads.gff3

| **Input** | PacBio-validation | Stem name for output files |
| --- | --- | --- |
|  | sample.initial-gene-models.gff | File containing gene models from an initial Augustus gene prediction |
|  | sample.aligned-PacBio-reads.gff3 | File containing GMAP aligned full-insert error corrected PacBio reads |
| **Output** | PacBio-validation.TranscriptStatus.txt | For each transcript: transcript ID, validation status, number confirming PacBio sequences, number of all sequences overlapping this transcript |
|  | PacBio-validation.ValidationDetails.txt | Detailed results on the evaluation of each overlapping Transcript-PacBio pair. |

**Description of the program**

PacBio sequences overlapping a predicted transcript are tested for the criteria below and are assigned a rank and label.

| **Criteria** | **Rank** | **Label** |
| --- | --- | --- |
| PacBio sequence encompasses transcript start and end positions | 6 | trStEnCovered |
| PacBio sequence encompasses the transcript’s translation start and stop codons | 5 | cdsStEnCovered |
| First transcript exon matches first PacBio exon | 4 | 5pOK |
| Concordant number of exons | 3 | numberExonsOK |
| Terminal transcript exon matches terminal PacBio exon | 2 | 3pOK |
| Concordant succession of exons, no internal exon is missing, start or end of transcript can be incomplete | 1 | successionOK |

To obtain a certain rank (e.g. rank 5, label cdsStEnCovered) all lower ranked criteria need to be met, too. At least two PacBio sequences need to confirm a transcript structure, else the transcript is labeled unconfirmed. A gene gets assigned the validation status of the transcript with the highest rank.

To extract transcripts confirmed by all overlapping PacBio sequences, run:

awk '($2=="trStEnCovered" && $3==$4){print $1}' PacBio-validation.TranscriptStatus.txt > PacBio-validation.TranscriptStatus.complete.ids

# or by at least 2 overlapping sequences run:

awk '($2=="trStEnCovered" && ($3==$4 || $3>=2)){print $1}' PacBio-validation.TranscriptStatus.txt > PacBio-validation.TranscriptStatus.completeMin2.ids

**Continuation**:

Training of Augustus on the validated transcripts. First, transcripts in gff format need to be converted into GenBank format using gff2gbSmallDNA.pl. The GenBank file will include flanking regions of a specified length. It is important to exclude other transcripts from the flanking regions, since flanking regions are used as intergenic training regions. First convert all initially predicted transcripts into GenBank and then extract the PacBio validated transcripts. Don’t use the “good” option in gff2gbSmallDNA.pl with the transcript IDs of the validated genes. This would only exclude these transcripts from flanking regions.
Then, remove redundant transcripts (on protein sequence level), transcripts with a CDS length not multiple of three and transcripts at scaffold borders. Thereafter, divide the remaining transcripts into a test and training set using randomSplit.pl. Finally run optimize_augustus.pl on either new parameters generated with new_species.pl or on existing parameters of a closely related species. For details and helper scripts see the [Augustus training documentation](http://bioinf.uni-greifswald.de/augustus/binaries/tutorial/training.html).

## II. Deriving gene models from PacBio full-insert reads

The provided script derives gene models directly from aligned full-insert error-corrected PacBio reads without using pre computed gene models

perl scripts/derive-gene-models-from-PacBio.pl PacBio-derived data/sample.aligned-PacBio-reads.gff3 data/sample.RefBeet-1.2.fna data/sample.intron-hints.gff

| **Input** | PacBio-derived | Stem name for output files |
| --- | --- | --- |
|  | sample.aligned-PacBio-reads.gff3 | File containing aligned full-insert error-corrected PacBio reads |
|  | sample.RefBeet-1.2.fna | Reference genome sequence in fasta-format |
|  | sample.intron-hints.gff | Intron hints (optional) |
| **Output** | PacBio-derived.gene-models.gff | Gene models in gff format |
|  | PacBio-derived.gene-models.fasta | transcript sequences in fasta format |
|  | PacBio-derived.gene-model-anchors.gff | Gene models as Augustus anchors in gff format |

**Description of the program**

Aligned PacBio reads are clustered based on their location and on intron boundaries.

The most abundant isoform per location confirmed by at least two PacBio reads is returned as a “PacBio-derived gene model”. The two reads that confirm an isoform need to have the same number and succession of exons, and same intron boundaries. Transcript boundaries are derived from the median start and stop positions of all aligned SMRT-sequences representing a selected isoform.

The PacBio derived gene models are also converted into Augustus anchors. Optionally intron evidence (e.g. from mRNA-seq data) can be provided to the script, which results in additional anchors derived from unclustered PacBio reads (singletons), if their intron boundaries could be confirmed by intron hints.

**Continuation**

Predict open reading frames with [TransDecoder](https://transdecoder.github.io/). In order to predict the longest possible open reading frame from PacBio-derived.gene-models.fasta add a sequence prefix containing stop codons in 3 reading frames (e.g. TAAATAAATAG). After running TransDecoder remove the prefix from the output and transfer the gene model coordinates to the reference genome using cdna_alignment_orf_to_genome_orf.pl (part of Transdecoder package). Use the PacBio derived gene models for training Augustus (for additional information see also section continuation under I.). The anchors (PacBio-derived.gene-model-anchors.gff) can be used directly for gene prediction with Augustus.

## III. RNA-seq noise reduction

Gene prediction accuracy is affected by noise in RNA-seq data, which is due to incompletely spliced mRNA molecules or rare isoforms. The provided filter script reduces noise to facilitate the correct prediction of the most abundant isoform per locus.

perl scripts/RNA-seq-noise-reduction.pl data/sample.intron-hints.gff data/sample.cov.wig sample.intron-hints.filt.gff sample.cov.filt.wig

| **Input** | sample.intron-hints.gff | intron hints generated with "blat2hints.pl --intronsonly" |
| --- | --- | --- |
|  | sample.cov.wig | wig file of aligned RNA-seq data generated with aln2wig |
| **Output** | sample.intron-hints.filt.gff | filtered intron hints |
|  | sample.cov.filt.wig | filtered wiggle file |

**Description of the program**

The default settings reduce the overall RNA-seq coverage by 10% of the local peak coverage (precisely 95 percentile, in 1-kbp windows). If the coverage difference between two overlapping intron hints (from gff file) is greater than 90% the intron hint with the lower coverage gets removed. The mRNA-seq coverage (from wig file) gets reduced within boundaries of introns by 50% of the adjacent exon coverage. Introns are considered if they are smaller or equal than 50 kbp in size and show a coverage drop of at least 50% at their exon-intron junction when comparing the coverage 10 bases upstream and downstream of each junction.

**Continuation**

Create hints from wiggle using Augustus wig2hints.pl (see [Augustus documentation](http://bioinf.uni-greifswald.de/augustus/binaries/readme.rnaseq.html) on RNA-seq integration). Merge filtered intron, exon hints with PacBio anchors from step II. Perform gene prediction with all hints.

## IV. Run Augustus with optimized parameters and hints

Improved gene predictions are calculated from optimized gene prediction parameters, optimized repeat and intron hint boni and maluses, noise reduced mRNA-seq exon and intron hints, intron anchors, repeat hints, PacBio anchors and optionally additional hints from Sanger ESTs or Roche/454 sequences.

The provided optimized beta_vulgaris prediction parameter folder contains parameters describing properties of gene features such as exons, introns, intergenic regions or UTRs. To employ these parameters in Augustus the folder needs to be placed into the Augustus species directory prior to executing Augustus.

cp -r data/beta_vulgaris $AUGUSTUS_CONFIG_PATH/species/

augustus --species=beta_vulgaris --hintsfile=data/sample.merged-hints.gff --extrinsicCfgFile=data/extrinsic.M.RM.E.W.RM.optimized.cfg --UTR=on --alternatives-from-evidence=true --allow_hinted_splicesites=atac data/sample.RefBeet-1.2.fna > optimized-gene-predictions.gff

| **Input** | --species=beta_vulgaris | Optimized Augustus prediction parameter folder |
| --- | --- | --- |
|  | --hintsfile=sample-merged-hints.gff | Concatenated hints file:   - Repeat hints - Noise reduced mRNA-seq exon and intron hints - Intron parts as anchors - PacBio anchors - Sanger ESTs, Roche/454 hints |
|  | --extrinsicCfgFile= extrinsic.M.RM.E.W.RM. optimized.cfg | Optimized repeat and intron hint bonus/ maluses |
|  | --UTR=on | Predict untranslated regions |
|  | --alternatives-from-evidence=true | Predict isoforms if supported y evidence |
|  | --allow_hinted_splicesites=atac | allow AT - AC splice sites if suggested by hints |
| **Output** | optimized-gene-predictions.gff | Improved Augustus gene models |

## V. Other scripts

**1. Select initial Augustus gene models for manual validation**

The Perl script parse_AUGUSTUS_gff3.pl creates a random gene set with a certain intron exon distribution and transcript hint coverage. This set can be used for manual verification of the underlying gene structures. For this purpose, transcript expression hint coverage of at least 85% was used.

perl scripts/parse_AUGUSTUS_gff3.pl -s 400 -f data/sample.RefBeet-1.1-gene-models.gff -c 85 > subsample.RefBeet-1.1-gene-models.gff

| **Input** | -s 400 | Size of output gene set |
| --- | --- | --- |
|  | -f sample.RefBeet-1.1-gene-models.gff | AUGUSTUS gene prediction file containing transcript coverage by expression hints |
|  | -c 85 | Include genes with a hint coverage greater then [-c] |
| **Output** | -c subsample.RefBeet-1.1-gene-models.gff | Filtered gene models |

**Algorithm description:**

Store all genes and number of associated exons with a transcript coverage of a certain coverage (85 used). Compute probabilities of exon and intron distribution for each represented exon number. Random selection of genes associated with a certain exon number applying the computed exon intron distribution to this gene set

**2. Transfer of stable gene identifiers between annotations**

The provided script assigns newly predicted RefBeet genes (BeetSet-2) a stable gene identifier of a previously predicted gene (RefBeet-1.1-genes), by comparing GMAP alignments of RefBeet-1.1-genes with gene model coordinates of BeetSet-2 genes.

perl scripts/transfer-stable-stable-identifier.pl data/sample.gene-IDs-RefBeet-1.1-prediction.txt data/sample.new-gene-models.gff data/sample.alignment-RefBeet-1.1-genes.gff

| **Input** | sample.gene-IDs-RefBeet-1.1-prediction.txt | Transcript IDs RefBeet-1.1 (previous prediction) and information on evidence support (1 = supported by evidence, 0 = not supported by evidence) |
| --- | --- | --- |
|  | sample.new-gene-models.gff | New Augustus gene models |
|  | sample.alignment-RefBeet-1.1-genes.gff | GMAP alignment of old transcripts |
| **Output** | old-new-ID-index.tab | Table containing the ID of the previous prediction and the new Augustus gene model ID to which the previous ID was transferred. |
